# The effects of an intervention program for promoting interorganizational network building between multidisciplinary agencies and community-based organizations: a cluster trial in Japan

**DOI:** 10.1186/1471-2458-12-178

**Published:** 2012-03-12

**Authors:** Hiroshi Murayama, Takuhiro Yamaguchi, Satoko Nagata, Sachiyo Murashima

**Affiliations:** 1 Research Team for Social Participation and Community Health, Tokyo Metropolitan Institute of Gerontology, 35-2 Sakae-cho, Itabashi-ku, Tokyo 173-0015, Japan; 2 Division of Biostatistics, Tohoku University Graduate School of Medicine, 2-1 Seiryo-machi, Aoba-ku, Sendai, Miyagi 980-8575, Japan; 3 Department of Community Health Nursing, Graduate School of Medicine, The University of Tokyo, 7-3-1 Hongo, Bunkyo-ku, Tokyo 113-0033, Japan

**Keywords:** Interorganizational network, Multidisciplinary agency, Community-based organization, Intervention program

## Abstract

**Background:**

Strengthening interorganizational relationships in the community has become an increasingly valued strategy for improving public health in recent years. However, no intervention strategy to foster an interorganizational network in the community has yet been devised. The purpose of this study was to examine the effects on members of an organization of an intervention program designed to promote interorganizational network building between multidisciplinary agencies and community-based organizations (CBOs).

**Methods:**

The program was conducted in Setagaya and Suginami wards, Tokyo, Japan, for staff of community comprehensive support centers (CCSCs), which are multidisciplinary organizations responsible for the support of the elderly. A cluster non-randomized design with a CCSC as a cluster unit (N = 47) was used. The intervention group comprised 20 centers and the control group 27 centers. Those 27 centers declined to participate in program sessions, but did participate through completing pre- and post-intervention surveys. In total, 158 staff members were eligible to participate in this study, 73 from the intervention group and 85 from the control group. Of the 73 members in the intervention group, 19 participated in the monthly program sessions, over a period of 10 months. Attendees participated in group discussions during the sessions. The effects of the intervention were examined by comparing three groups (attendees and non-attendees of the program from the intervention group, and the control group) and between two groups (intervention group and control group).

**Results:**

We found no significant difference in any outcome between the intervention group and the control group. However, among the three groups, a significant effect was found in the recognition of knowledge and skills for building networks with CBOs. Recognition of knowledge and skills increased significantly among the attendees compared to non-attendees in the intervention group and the control group. In addition, there was a significant effect, particularly on those with relatively low baseline scores, for the recognition of knowledge and skills.

**Conclusions:**

The tested intervention proved effective for attendees regarding their recognition of knowledge and skills for promoting interorganizational network building with CBOs.

## Background

The growth of medical and nursing care costs has been recognized as a social problem in Japan today because of its aging population, which is aging more rapidly than other nations. It is apparent that a single health and welfare agency cannot address this issue and that various kinds of organizations must work together. Strengthening interorganizational collaboration in the community to leverage both social and material resources for problem solving has become an increasingly valuable strategy to improve public health in recent years [1,2]. An interorganizational network can enhance collaborative problem solving by pooling knowledge and insights, sharing resources, and seeking common solutions. Other major benefits of collaboration include the ability to deliver services more efficiently and effectively by minimizing duplication and providing services that meet the multiple needs of clients; the potential to maximize power and influence by combining forces; and the shared responsibility across organizations for complex or controversial issues [3-7]. A positive attitude toward collaboration, mutual shared goals, regular funding, and resources such as staff, time and expertise to maintain the network are other factors associated with fostering organizational networks [3,8,9]. These factors have important implications when evaluating the current coalitions or alliances and when discussing improvements in the present systems. In reality an intervention strategy to foster interorganizational networks in the community is needed, but has yet to be devised.

Japan has community comprehensive support centers (CCSCs), which function as hubs for the provision of healthcare and welfare for the elderly in the community. These centers were established in 2006 in response to the rapidly aging population throughout the country. They are multidisciplinary organizations in which three professions (public health nurses (or registered nurses), certified social workers and care managers) work together to provide support to the elderly. One important function of CCSCs is to build an organizational network with community-based organizations (CBOs). Local residents, rather than professionals, run these CBOs and they are expected to provide informal, voluntary and practical services for the elderly. CBOs are, for example, neighborhood associations and district welfare commissions. CCSCs and CBOs often share information about the elderly who need to be looked after or supported, and discuss issues in community support through the organizational networks. CCSCs are expected to connect the elderly to these CBOs and then share with them the provision of the care to the elderly. However, staff in CCSCs have found it difficult to build networks with CBOs and wanted to know how to form such networks because they had little knowledge or experience in network building with CBOs [10].

Against such a background, we developed an intervention program for staff of the CCSCs to promote interorganizational network building between those multidisciplinary agencies and CBOs [10]. This program, based on Social Cognitive Theory (SCT) [11-13], aimed to enhance recognition of the importance of networking, to provide networking skills development, and to encourage network building with CBOs. SCT has provided a foundation for many intervention programs. The theoretical concept is reciprocal determinism, which maintains that there is constant interaction among personal (including cognition), behavioral and environmental factors [11]. For example, as the recognition and the motivation of an individual in the CCSC for interorganizational network building with CBOs increases, he/she will engage positively in the work of network building. This can also motivate the other staff in the same CCSC to get involved in that work, and as a result, the organizational environment and system of the CCSC will change. Therefore, the study focused on knowledge of and attitude toward interorganizational network building with CBOs (a personal factor), involvement in interorganizational network building with CBOs, such as negotiation with CBOs and provision of joint care (a behavioral factor) at an individual level, and enhancement of momentum for network building with CBOs by the entire CCSC, such as frequent staff meetings concerning network building in the organization (an environmental factor at the organizational level).

The purpose of this study was to examine the effects of the intervention program provided for members of CCSCs. We proposed two hypotheses. First, the attendees at the program will experience a significant improvement in recognition of the value of building networks with CBOs and increased involvement in the work of network building with CBOs in comparison with non-attendees (individual personal and behavioral factors). Second, the entire staff of the CCSC to which the attendee at the program belonged will also experience the same advantages to a significant degree in comparison with the staff of a CCSC without an attendee (an organizational environmental factor). In this study we defined interorganizational network building between the CCSC and the CBOs as follows: to build any formal and organization-conscious connection or relationship with CBO members. This excluded any informal and personal connections or relationships.

## Methods

### Setting and participants

The study was conducted in Setagaya and Suginami, two neighboring wards in downtown western Tokyo. These wards are predominantly residential. In April 2007, Setagaya had a population of 804,699 (385,768 male and 418,931 female), a population density of 13,854 people per square kilometer and 413,404 households. People aged 65 years and over (elderly) made up 17.1% of the population. In April 2009, Suginami had a population of 520,957 (251,465 male and 269,492 female), a population density of 15,313 people per square kilometer, 286,115 households and 18.7% of the population was elderly. The percentages of elderly in Setagaya and Suginami have been increasing rapidly, from 16.0% (Setagaya) and 16.8% (Suginami) in 2000, and are estimated to reach 25.8% (Setagaya) and 25.5% (Suginami) by the year 2030 [14].

Setagaya has 27 CCSCs and Suginami has 20. Participants in this program were staff of these CCSCs. Staff completed self-administered questionnaire surveys pre and post intervention.

### Study design and procedure

**Figure 1:**
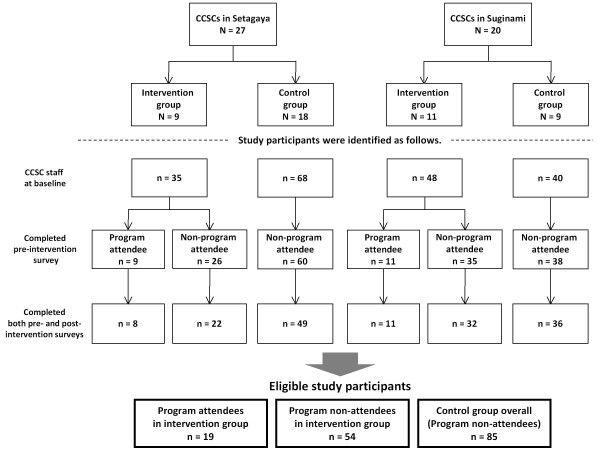
**Flow diagram of study participants**. The process of allocation of the study participants to intervention and control groups is shown.

This study adopted a cluster non-randomized design with a CCSC as a cluster unit. Figure 1 is a flow diagram of the study participants in each ward. First, we invited each CCSC to join the program. In Setagaya, nine of the 27 CCSCs applied for the program as did 11 of the 20 CCSCs in Suginami. These CCSCs were assigned to the intervention group and those that did not apply for the program were assigned to the control group; thus, the intervention group consisted of 20 CCSCs and the control group consisted of 27. Second, CCSCs in the intervention group were asked to nominate one staff member to attend all the program sessions. Most staff who participated in the program were chosen by the managers of each CCSC.

The program ran in Setagaya in 2007 and in Suginami in 2009. In Setagaya, in April 2007, a pre-intervention survey of the 103 professionals belonging to the 27 CCSCs (35 in the intervention group and 68 in the control group) was conducted after providing information about the study. A post-intervention survey was conducted in March 2008. In Suginami, in April 2009, the pre-intervention survey of the 88 professionals belonging to the 20 CCSCs (48 in the intervention group and 40 in the control group) was conducted after providing information about the study. The post-intervention survey was conducted in March 2010.

The participants were subsequently divided into three groups: program attendees (intervention group), program non-attendees (intervention group) and the control group. The first hypothesis was tested by comparing the outcomes from the three groups (1 = program attendees in the intervention group; 2 = program non-attendees in the intervention group; 0 = the control group). The second one was tested by comparing the intervention group as a whole with the control group (1 = the intervention group; 0 = the control group).

### Ethical considerations

The Ethics Committee of the Faculty of Medicine at The University of Tokyo granted approval for the study. At the time of distribution of the pre-program questionnaire, potential participants were informed of the purpose and methods of this study and that their participation was optional. This statement, a guarantee of anonymity, and descriptions of other aspects of the cooperation requested were attached to the questionnaire. Return of the questionnaire was deemed to be consent to participate in the study. Individual participants were allocated an identifying code not known to the data manager.

### Intervention program

The authors developed the intervention program that they delivered over 10 monthly sessions [10]. The themes and objectives of the sessions were based on the stages of the coalition development model proposed by Florin et al. [15]. The model explains the ongoing development of organizational collaborative relationships in stages. Because this study focused on interorganizational networks between CCSC and CBOs, session themes included "review of past activities," "understanding the significance of building networks with CBOs," "integrating the aims and visions of staff," and "learning ways to build networks with CBOs." Participants shared opinions and experiences about network building with CBOs in group discussions at each session.

"Review of past activities" was designed to clarify the strengths and weaknesses of past network building efforts with CBOs by the attendees' CCSCs. Those attending identified the types of CBOs they had contacted and described the nature of their connections. "Understanding the significance of building networks with CBOs" aimed to establish the basic motivation for building interorganizational networks with CBOs. Attendees discussed why they built networks with CBOs and when they considered it necessary to build networks. "Integrating the aims and visions of staff" discussed ways to build consensus, to enhance the recognition of the importance of network building and to create ideal situations in the community by building networks with CBOs. In "Learning ways to build networks with CBOs", the attendees shared their existing strategies in building networks with CBOs according to the types of CBOs.

The attendees evaluated the content of the program and indicated a high level of satisfaction with it [16]. Their self-efficacy based on the SCT increased after completing the program [16].

### Measures

#### Outcome variables

Cognitive and behavioral dimensions were the measured outcomes. The cognitive dimension included recognition of the knowledge and skills used in building networks with CBOs, the ease of working with CBOs within existing networks, and the importance of building networks with CBOs. The first cognitive dimension was assessed using the subscale of "Knowledge and Skills" from the Social Worker Empowerment Scale [17]. We revised the wording of the items to fit into the context of this study. This subscale consisted of nine items with responses on a 5-point Likert scale. We conducted a factor analysis using baseline data, and confirmed the nine items loading into a single factor. Scores were summed (ranging from 9 to 45): the higher the score, the greater the recognition of knowledge and skills by the respondents. Cronbach's alpha was 0.83 as calculated from baseline data. One item was used to assess each of the second and third cognitive dimensions. The statements were "I think that the work at the CCSC will become easier through networking with CBOs" and "I think that building a network with CBOs is important to my work at the CCSC." These items were scored on a 6-point Likert scale (6 = strongly agree; 5 = agree; 4 = somewhat agree; 3 = somewhat disagree; 2 = disagree; 1 = strongly disagree). The focus for the behavioral dimension related to the involvement in interorganizational network building with CBOs. The statement was "What is the current percentage of your entire work at the CCSC that is related to network building with CBOs?" Answers could range from 0 to 100%.

#### Demographic variables

Data were collected on age, sex (1 = male; 2 = female), educational level (1 = high school graduate; 2 = Junior college/vocational school graduate; 3 = college graduate), years of experience in community-based clinical practice, years of experience in clinical practice in the current catchment area of the CCSC, type of profession, and average weekly working hours (1 = under 30 hours; 2 = 30-39 hours; 3 = 40-49 hours; 4 = 50-59 hours; 5 = 60-69 hours; 6 = 70 hours or more). Type of profession was categorized as "public health nurse and registered nurse" (= 1), "certified social worker" (= 2) or "care manager" (= 3).

### Statistical analysis

One of the purposes of this trial was to compare the recognition of the value of building networks with CBOs and the involvement in the work of network building with CBOs among the three groups; program attendees in the intervention group, program non-attendees in the intervention group, and the control group (at personal and behavioral level). The ratio of the number included in each group was designed as a one-three-four, on the assumption that CCSCs are equally allocated to intervention and control groups, a CCSC (a unit of cluster) composed of four staff on average, with one staff member attending all the program sessions from each CCSC in the intervention group. We postulated that a significant effect would be to detect a 15% difference in the score of the recognition of knowledge and skills, which was one of the outcomes measured in this study. As a consequence, a total sample size of 176 staff was projected to provide a power of 80% with α = 0.05 to detect a 15% difference between the three groups, assuming 0.05 of intra-class correlation coefficient and 10% dropout rate. This size was sufficient to test the other hypothesis to examine the difference between two groups of the intervention group and the control group (at organizational environmental level).

In the first stage of our analysis we tested the demographic variables and baseline scores of the outcome variables to assess comparability among the groups. Each CCSC was a cluster unit and was allocated to either the intervention or the control group. We used generalized estimating equations (GEE) taking into account the extra component of variation due to the nested design. In this study, we adopted compound symmetry as the working correlation structure.

Second we assessed the main effects of the intervention on the outcome variables using GEE after adjusting for the baseline scores of the outcome variables that differed significantly among the groups at baseline. When the GEE showed a significant difference, the intervention had had a significant effect on the outcome variable.

Third we assessed the interactions of the intervention according to the baseline scores for the outcome variables by adding interactions to the analysis of the main effect using GEE in order to examine in more detail the effects of the intervention program. A statistically significant interaction meant that the intervention had different effects according to the baseline score for the outcome variables. When we found a significant interaction, the participants were divided into two groups according to the median of the baseline score of the outcome variable and the interactions of the intervention were analyzed again.

Statistical significance was set as *p *< 0.05 with a two-tailed test. The statistical analyses were performed using SAS ver. 9.1.

## Results

### Characteristics of the intervention group and the control group

In Setagaya, 30 staff members in the intervention group and 49 in the control group were eligible as study participants. Nine members of the intervention group attended the program, but one dropped out before completion because of maternity leave. In Suginami, 43 members in the intervention group and 36 in the control group were eligible as study participants. Eleven members of the intervention group attended the program. In total, 73 members of the intervention group and 85 members of the control group participated in this study through completion of the pre and post intervention surveys. Of the 73 members of the intervention group 19 attended the program and 54 did not (Figure 1).

Table 1 shows the demographic characteristics of the study participants. Working hours differed significantly between the intervention and control groups, with control group staff working more hours than intervention group staff. Among the three groups (attendees in intervention group, non-attendees in intervention group and control group), there was a significant difference in working hours and a marginally significant difference in years of experience in community-based clinical practice. Table 2 shows the baseline scores for the outcome variables. No differences in any variable between the two groups or among the three groups were observed. No significant differences were found in demographic characteristics and baseline scores for the outcomes between the respondents from Setagaya and Suginami (data not shown).

**Table 1:** Demographic characteristics of study participants

		Intervention group			Control group	*p*-value	
			
		(a) Overall n = 73	(b) Attendees n = 19	(c) Non- attendees n = 54	(d) Overall n = 85	Between 2 groups (a) vs. (d)	Among 3 groups (b), (c) vs. (d)
Sex	Female	64 (84.2)	14 (73.7)	50 (87.7)	70 (80.5)	0.533	0.325
Age		44.2 ± 9.6	42.8 ± 9.0	44.7 ± 9.6	42.3 ± 9.9	0.220	0.368
Educational level	College graduate	42 (55.3)	12 (63.2)	30 (52.6)	50 (58.8)	0.276	0.477
	Junior college/vocational school graduate	6 (7.9)	0 (0.0)	6 (10.5)	16 (18.8)		
	High school graduate	28 (36.8)	7 (36.8)	21 (36.8)	19 (22.4)		
Years of experience in clinical practice in the community		6.9 ± 5.6	9.9 ± 7.8	5.9 ± 4.2	5.8 ± 4.1	0.167	0.074
Years of experience in clinical practice in catchment area of CCSC to which participants belonged		4.0 ± 3.8	5.6 ± 6.0	3.5 ± 2.5	3.5 ± 2.8	0.280	0.310
Type of profession	Public health nurse/Registered nurse	20 (26.3)	6 (31.6)	14 (24.6)	19 (21.8)	0.671	0.784
	Certified social worker	25 (32.9)	7 (36.8)	18 (31.6)	34 (39.1)		
	Care manager	31 (40.8)	6 (31.6)	25 (43.9)	34 (39.1)		
Working hours/week	Under 30 hours	5 (6.7)	0 (0.0)	5 (8.9)	1 (1.1)	0.036	0.009
	30-39 hours	9 (12.0)	1 (5.3)	8 (14.3)	9 (10.3)		
	40-49 hours	46 (61.3)	12 (63.2)	34 (60.7)	48 (55.2)		
	50-59 hours	11 (14.7)	3 (15.8)	8 (14.3)	22 (25.3)		
	60-69 hours	2 (2.7)	1 (5.3)	1 (1.8)	2 (2.3)		
	70 hours or more	2 (2.7)	2 (10.5)	0 (0.0)	5 (5.7)		

CCSC = community comprehensive support center.

Values represent n (%) or mean ± SD.

**Table 2:** Baseline scores for outcome variables

	Intervention group			Control group	*p*-value	
			
	(a) Overall n = 73	(b) Attendees n = 19	(c) Non- Attendees n = 54	(d) Overall n = 85	Between 2 groups (a) vs. (d)	Among 3 groups (b), (c) vs. (d)
Recognition of knowledge and skills for building networks with CBOs (range 9-45)	20.8 ± 4.8	22.0 ± 6.2	20.3 ± 4.2	21.5 ± 4.9	0.345	0.249
Recognition of ease of working through networking with CBOs (range 1-6)	5.0 ± 0.8	5.1 ± 0.7	4.9 ± 0.9	4.8 ± 0.8	0.272	0.268
Recognition of importance of building networks with CBOs (range 1-6)	5.1 ± 0.7	5.4 ± 0.6	5.1 ± 0.7	5.0 ± 0.8	0.331	0.092
Percentage of working hours to build network with CBOs (%) (range 0-100)	19.5 ± 19.0	21.6 ± 17.5	18.7 ± 19.5	16.1 ± 15.4	0.216	0.359

CBO = community-based organization.

Values represent mean ± SD.

### Main effect of the intervention

We found no significant difference in any outcome between the intervention and control groups (Table 3). However, among the three groups, a significant effect was found in recognition of knowledge and skills for building networks with CBOs (*χ*^2 ^= 12.74, *p *= 0.002), but not on the behavioral outcome (Table 4). The recognition of knowledge and skills by attendees increased significantly compared to non-attendees in the intervention group and the control group.

**Table 3:** Main effects of the intervention on the outcomes: comparison between intervention group and control group

	(a) Intervention group overall n = 73	(d) Control group overall n = 85	**χ**^**2**^	*p*-value
Recognition of knowledge and skills for building networks with CBOs				
At baseline	20.8 ± 4.8	21.5 ± 4.9	1.64	0.201
Post intervention	22.6 ± 5.1	22.6 ± 4.6		
LS mean ± SE	23.6 ± 0.8	22.8 ± 0.7		
Recognition of ease of working through networking with CBOs				
At baseline	5.0 ± 0.8	4.8 ± 0.8	0.60	0.440
Post intervention	5.0 ± 0.8	4.9 ± 0.9		
LS mean ± SE	4.9 ± 0.1	4.8 ± 0.1		
Recognition of importance of building networks with CBOs				
At baseline	5.1 ± 0.7	5.0 ± 0.8	0.04	0.838
Post intervention	5.0 ± 0.7	5.0 ± 0.7		
LS mean ± SE	5.0 ± 1.1	5.0 ± 1.1		
Percentage of working hours building networks with CBOs (%)				
At baseline	19.5 ± 19.0	16.1 ± 15.4	0.38	0.540
Post intervention	17.8 ± 13.4	17.2 ± 12.7		
LS mean ± SE	18.6 ± 2.4	19.8 ± 2.3		

CBO = community-based organization. LS mean = least square mean.

Values represent mean ± SD.

Adjusted by sex, age, years of experience in clinical practice in the community, type of profession, working hours and baseline data on outcome variables.

**Table 4:** Main effects of the intervention on the outcomes: comparison among the three groups

	Intervention group		Control group			
					
	(b) Attendees n = 19	(c) Non-attendees n = 54	(d) Overall n = 85	**χ **^**2**^	*p*-value	Post hoc
Recognition of knowledge and skills for building networks with CBOs						
At baseline	22.0 ± 6.2	20.3 ± 4.2	21.5 ± 4.9	12.74	0.002	(b) vs. (c): *p *= 0.002
Post intervention	26.0 ± 3.8	21.4 ± 5.1	22.6 ± 4.6			(b) vs. (d): *p *< 0.001
LS mean ± SE	25.8 ± 1.0	22.5 ± 0.9	22.6 ± 0.7			(c) vs. (d): *p *= 0.854
Recognition of ease of working through networking with CBOs						
At baseline	5.1 ± 0.7	4.9 ± 0.9	4.8 ± 0.8	2.82	0.244	(b) vs. (c): *p *= 0.117
Post intervention	5.3 ± 0.8	4.9 ± 0.8	4.9 ± 0.9			(b) vs. (d): *p *= 0.108
LS mean ± SE	5.1 ± 0.2	4.8 ± 0.2	4.8 ± 0.1			(c) vs. (d): *p *= 0.915
Recognition of importance of building networks with CBOs						
At baseline	5.4 ± 0.6	5.1 ± 0.7	5.0 ± 0.8	2.94	0.231	(b) vs. (c): *p *= 0.088
Post intervention	5.3 ± 0.6	4.9 ± 0.7	5.0 ± 0.7			(b) vs. (d): *p *= 0.154
LS mean ± SE	5.2 ± 0.2	5.0 ± 0.1	5.0 ± 0.1			(c) vs. (d): *p *= 0.612
Percentage of working hours building networks with CBOs (%)						
At baseline	21.6 ± 17.5	18.7 ± 19.5	16.1 ± 15.4	2.77	0.250	(b) vs. (c): *p *= 0.114
Post intervention	22.5 ± 11.7	16.2 ± 13.9	17.2 ± 12.7			(b) vs. (d): *p *= 0.366
LS mean ± SE	22.2 ± 3.1	17.0 ± 2.7	19.5 ± 2.4			(c) vs. (d): *p *= 0.241

CBO = community-based organization. LS mean = least square mean.

Values represent mean ± SD.

Adjusted by sex, age, years of experience in clinical practice in the community, type of profession, working hours and baseline data on outcome variables.

### The effect of the intervention according to baseline outcome scores

**Figure 2:**
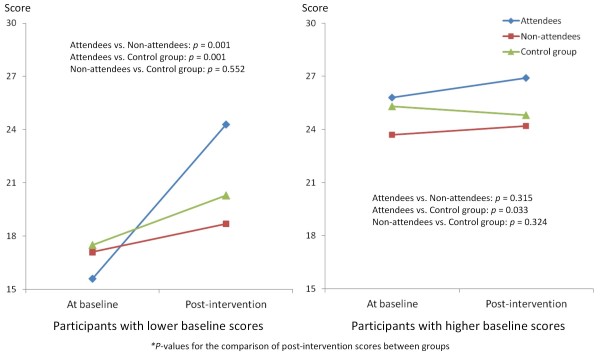
**Effect of the intervention on the recognition of knowledge and skills for building networks with community-based organizations**. The outcome of the intervention with regard to recognition of knowledge and skills for building networks with CBOs among three groups, each divided according to baseline scores (high and low) is shown.

**Figure 3:**
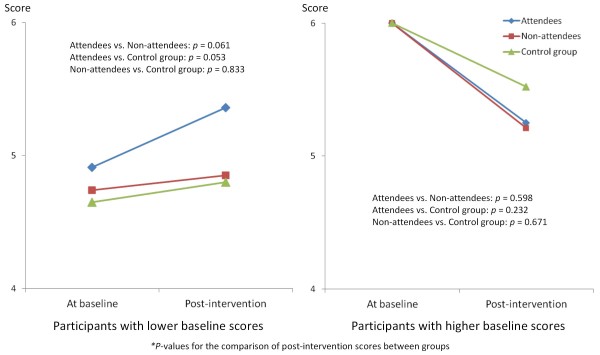
**Effect of the intervention on recognition of the importance of building networks with community-based organizations**. The outcomes of the intervention with regard to recognition of the importance of building networks with CBOs among three groups, each divided according to baseline scores (high and low) are shown.

We conducted an additional analysis to clarify details of the effect of the intervention. As indicated in Table 5, significant interactions could be found between baseline scores and the three groups for the recognition of knowledge and skills for building such networks (*χ*^2 ^= 6.18, *p *= 0.046) and the importance of building networks with CBOs (*χ*^2 ^= 6.06, *p *= 0.048). To examine in more detail the intervention effect by differences in the baseline outcome scores, we divided the participants into two groups based on the median of these two baseline outcome scores. On recognition of knowledge and skills, for those with lower baseline scores, we found that attendees had a significantly higher score than non-attendees in the intervention group and the control group. Among those with higher baseline scores, attendees had a significantly higher score than the control group (Figure 2). Similar results were found for the participants with lower baseline scores for the recognition of importance of building networks with CBOs (Figure 3).

**Table 5:** Interaction of baseline score of outcome variables and the groups with the outcomes

	Intervention group		Control group	Baseline × groups	
			
	(b) Attendees n = 19	(c) Non-attendees n = 54	(d) Overall n = 85	***χ ***^**2**^	*p*-value
Recognition of knowledge and skills for building networks with CBOs					
LS mean ± SE	26.1 ± 0.9	22.6 ± 0.8	22.6 ± 0.7	6.18	0.046
Recognition of ease of working through networking with CBOs					
LS mean ± SE	5.1 ± 0.2	4.8 ± 0.2	4.8 ± 0.1	0.18	0.916
Recognition of importance of building networks with CBOs					
LS mean ± SE	5.4 ± 0.2	5.0 ± 0.1	5.0 ± 0.1	6.06	0.048
Percentage of working hours building networks with CBOs (%)					
LS mean ± SE	23.0 ± 3.2	16.8 ± 2.7	19.5 ± 2.4	0.93	0.629

CBO = community-based organization. LS mean = least square mean.

Adjusted by sex, age, years of experience in clinical practice in the community, type of profession, working hours and baseline data on outcome variables.

## Discussion

From our results, we concluded that the intervention led to a significant and positive effect for individual participants in terms of interorganizational network building between the multidisciplinary agencies and CBOs. The scores regarding the recognition of the knowledge and skills required for network building with CBOs for program attendees increased significantly compared to those for non-attendees in the intervention group and the control group. This was substantially greater among attendees compared with those who did not attend the program. Our previous study showed that CCSC staff felt they had little opportunity to learn exactly how to build an interorganizational network with CBOs [10]. The importance of having expertise in maintaining collaborative interorganizational networks has been suggested in previous research [1]. The program we presented followed the developmental model for organizational collaborative relationships proposed by Florin et al. [15], and the attendees were able to learn the essential steps in building an interorganizational network with CBOs. This contributed to an increase in knowledge and skills related to interorganizational network building with CBOs.

Another outcome of the recognition of importance of building networks with CBOs on the cognitive dimension had ceiling effects at baseline scores. Therefore, we could not confirm the positive effect of this outcome. The other outcome of recognition of ease of working through networking with CBOs also had a high score at baseline already. The improvement of these outcomes must be measured in a future study.

In an additional analysis after dividing the participants into two groups according to baseline outcome scores, we found a clearly significant effect, particularly on program attendees who had a lower baseline score, for recognition of knowledge and skills. This indicates that attendees of the program, who at baseline felt deficient in this area, considered attendance had increased their knowledge and skills in building an interorganizational network. Similarly, the attendees of the program with lower baseline scores on recognition of the importance of building an interorganizational network with CBOs came to recognize the importance of this after the intervention to a greater degree than those who did not attend. These two outcomes correspond to behavioral capability and expectancy, which are constructs of the SCT [11-13]. The attendees could obtain knowledge and skills in relation to a behavior and expect that network building with CBOs was likely to be improved by participating in the program. The additional analyses showed that an advantage of this intervention program would be raising the level of the recognition of the importance of building interorganizational networks with CBOs as well as recognition of the knowledge of skills required to do so. This outcome could be expected particularly among the program attendees who had lower baseline scores for these outcomes.

However, there was no significant difference in the behavioral variable among the three groups. No positive effects of the program on the overall intervention group compared with the control group were found in contrast to our hypothesis. The lack of, or otherwise modest, effects of the intervention on the hypothesized outcomes may be explained in several ways. It may be that an intervention of a longer duration is needed to generate greater behavioral and environmental changes in the outcomes. Furthermore, it takes time and a substantial commitment of CCSC staff to build organizational relationships with CBOs. Therefore, we should monitor the effect of our program with a long-term follow-up survey. Bandura explained the importance of the interaction among personal, behavioral and environmental components to make behavioral changes as a construct of reciprocal determinism [11-13]. At the same time, he emphasized that a change in one component has implications for the other two components [11]. That is, improvements in both the recognition of knowledge and skills as well as the importance of network building with CBOs through attendance at the program possibly trigger changes in behavioral and environmental factors. These individuals with growing recognition could increase their actual involvement in interorganizational network building (a behavioral change), and inspire the other staff of the CCSC to get involved in network building with CBOs (an environmental change). Therefore, to have an impact on behavioral and environmental outcomes a more intensive intervention must be designed, and follow-up monitoring of study participants must be conducted to evaluate the medium- and long-term effects of the program on these outcomes.

Overall, we clearly demonstrated positive effects of the intervention only on the attendees of the program. To our knowledge, this is the first report of a program for promoting interorganizational network building between multidisciplinary agencies and CBOs. Several studies have described interorganizational collaborative relationships using network analysis [8,18-20] and described the development of a theoretical model for the formation of collaborative relationships between organizations [3,9,15]. However, in practical situations, those working in the community, such as public health nurses and care workers in CCSCs, had difficulty in building networks with CBOs and desired to learn of ways of building such networks [10]. We believe that this intervention program can encourage staff of multidisciplinary agencies such as CCSCs to engage in the work of building networks with CBOs in the community.

We must consider several limitations of the present study. First, the study had a cluster non-randomized design. At baseline, there was a significant difference between the intervention and control groups in that the control group staff had more working hours than those in the intervention group. Staff of CCSCs in the control group might have had less time to participate in the program. We used this variable as a covariate in the analysis to adjust for such a difference between the intervention and control groups. A cluster randomized design is more robust and suitable than a non-randomized design in a community-based intervention [21]. Therefore, a future study should be a cluster randomized trial if possible. Second, the attendees of the program were not randomly selected from each CCSC in the intervention group. They might have had an interest in and desire to engage in activities of organization network building. Therefore, selection bias would exist. Third, all subjects of this study were not blind to the group's assignment. This may have affected the results. Fourth, this intervention program focused on the interorganizational network between multidisciplinary agencies and CBOs, but the participants were only the staff of multidisciplinary agencies (CCSCs). To promote effective interorganizational networks between these two types of organizations, the collaboration between both organizations would be essential. The program should be modified to include members from the CBOs. Fifth, the program was carried out at two different times (2007 and 2009), although the same program was implemented. Therefore, the period effect may affect the results of this study. Finally, this trial was conducted only in an urban area in Japan. The community context, such as community politics, history, norms and values, can influence coalition membership [22]. Therefore, it is difficult to generalize this study's findings to areas with other characteristics (e.g. rural areas) and to other countries.

## Conclusions

We examined the effects on members of an organization of an intervention program to promote interorganizational network building between multidisciplinary agencies and CBOs. The intervention increased the program attendees' recognition of the knowledge and skills required for promoting interorganizational network building with CBOs. This study provides practical evidence of a strategy to foster the development of interorganizational networks in the community, especially between multidisciplinary agencies and CBOs. Further work in this area could focus on the development of a comprehensive study involving both multidisciplinary agencies and CBOs.

## Competing interests

The authors declare that they have no competing interests.

## Authors' contributions

HM conceived the study, performed data collection and data analysis, and drafted the manuscript. TY assisted in the statistical analysis and contributed to the interpretation of the results. SN and SM conceived the study and contributed to the interpretation of the results. All authors read and approved the final manuscript.

## Pre-publication history

The pre-publication history for this paper can be accessed here:

http://www.biomedcentral.com/1471-2458/12/178/prepub
